# Crystal structure of β-d,l-allose

**DOI:** 10.1107/S2056989015000353

**Published:** 2015-01-31

**Authors:** Tomohiko Ishii, Tatsuya Senoo, Taro Kozakai, Kazuhiro Fukada, Genta Sakane

**Affiliations:** a Department of Advanced Materials Science, Faculty of Engineering, Kagawa University, 2217-20 Hayashi-cho, Takamatsu, Kagawa 761-0396, Japan; b Department of Applied Biological Science, Faculty of Agriculture, Kagawa University, 2393 Ikenobe, Kagawa 761-0795, Japan; c Department of Chemistry, Faculty of Science, Okayama University of Science, 1-1 Ridaicho, Kita-ku, Okayama 700-0005, Japan

**Keywords:** crystal structure, racemic compound, rare sugar, O—H⋯O hydrogen bonding

## Abstract

The title compound, C_6_H_12_O_6_, a C-3 position epimer of glucose, was crystallized from an equimolar mixture of d- and l-allose. It was confirmed that d-allose (l-allose) formed β-pyran­ose with a ^4^ *C*_1_ (^1^ *C*_4_) conformation in the crystal. In the crystal, molecules are linked by O—H⋯O hydrogen bond, forming a three-dimensional framework. The cell volume of the racemic β-d,l-allose is 739.36 (3) Å^3^, which is about 10 Å^3^ smaller than that of chiral β-d-allose [*V* = 751.0 (2) Å^3^].

## Related literature   

For the crystal structure of the chiral β-d-allose, see: Kroon-Batenburg *et al.* (1984 ▸). For the crystal structure of racemic d,l-arabinose, see: Longchambon *et al.* (1985 ▸) and of chiral l-arabinose, see: Takagi & Jeffrey (1977 ▸). For the synthesis of chiral d- or l-allose, see: Menavuvu *et al.* (2006 ▸); Morimoto *et al.* (2006 ▸, 2013 ▸); Shimonishi & Izumori (1996 ▸).
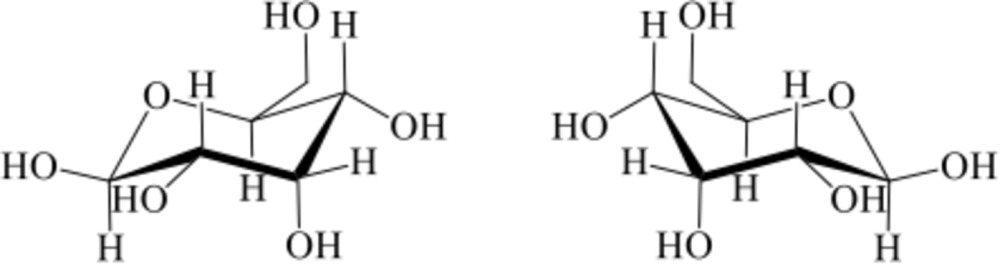


## Experimental   

### Crystal data  

C_6_H_12_O_6_
*M* *_r_* = 180.16
Monoclinic, 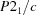

*a* = 4.98211 (10) Å
*b* = 12.5624 (3) Å
*c* = 11.8156 (3) Å
β = 91.1262 (14)°
*V* = 739.36 (3) Å^3^
*Z* = 4
Cu *K*α radiation
μ = 1.29 mm^−1^
*T* = 295 K
0.10 × 0.10 × 0.10 mm

### Data collection  

Rigaku R-AXIS RAPID diffractometer
Absorption correction: multi-scan (*ABSCOR*; Higashi, 1995 ▸) *T*_min_ = 0.687, *T*_max_ = 0.879
12963 measured reflections
1350 independent reflections
1232 reflections with *F* ^2^ > 2σ(*F* ^2^)
*R*_int_ = 0.075

### Refinement  

*R*[*F* ^2^ > 2σ(*F* ^2^)] = 0.037
*wR*(*F* ^2^) = 0.102
*S* = 1.07
1350 reflections
115 parameters
H-atom parameters constrained
Δρ_max_ = 0.37 e Å^−3^
Δρ_min_ = −0.22 e Å^−3^

### 

Data collection: *RAPID-AUTO* (Rigaku, 2009 ▸); cell refinement: *RAPID-AUTO*; data reduction: *RAPID-AUTO*; program(s) used to solve structure: *SIR2008* in *Il Milione* (Burla *et al.*, 2007 ▸); program(s) used to refine structure: *SHELXL2013* (Sheldrick, 2015 ▸); molecular graphics: *CrystalStructure* (Rigaku, 2010 ▸); software used to prepare material for publication: *CrystalStructure*.

## Supplementary Material

Crystal structure: contains datablock(s) General, I. DOI: 10.1107/S2056989015000353/is5386sup1.cif

Structure factors: contains datablock(s) I. DOI: 10.1107/S2056989015000353/is5386Isup2.hkl

**Fig. 1.**
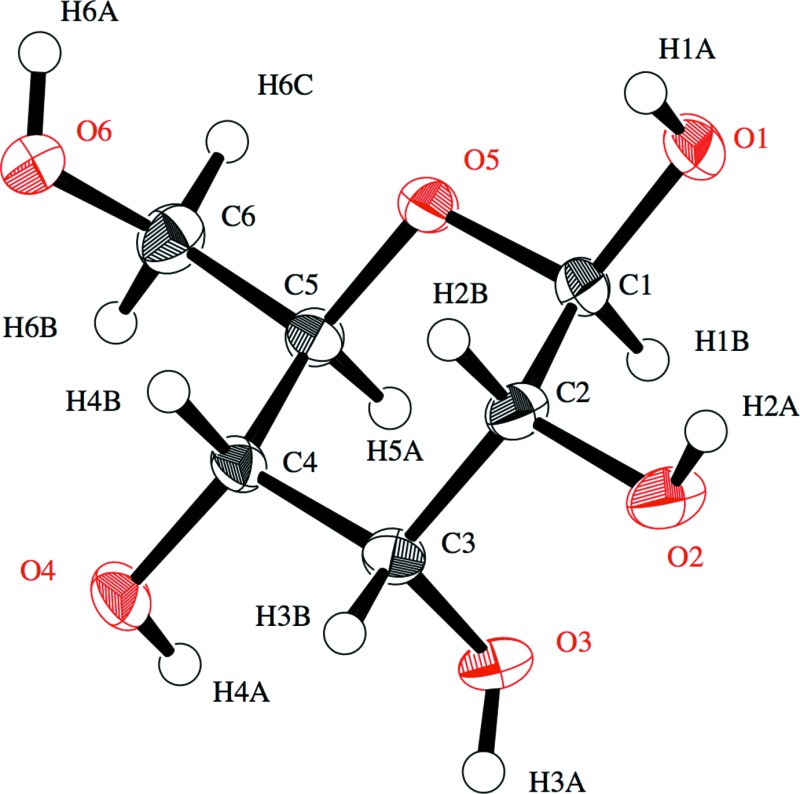
ORTEP view of the title compound with the atom-labeling scheme. The thermal ellipsoids of all non-hydrogen atoms are drawn at the 50% probability level. H atoms are shown as small spheres of arbitrary radius.

**Fig. 2.**
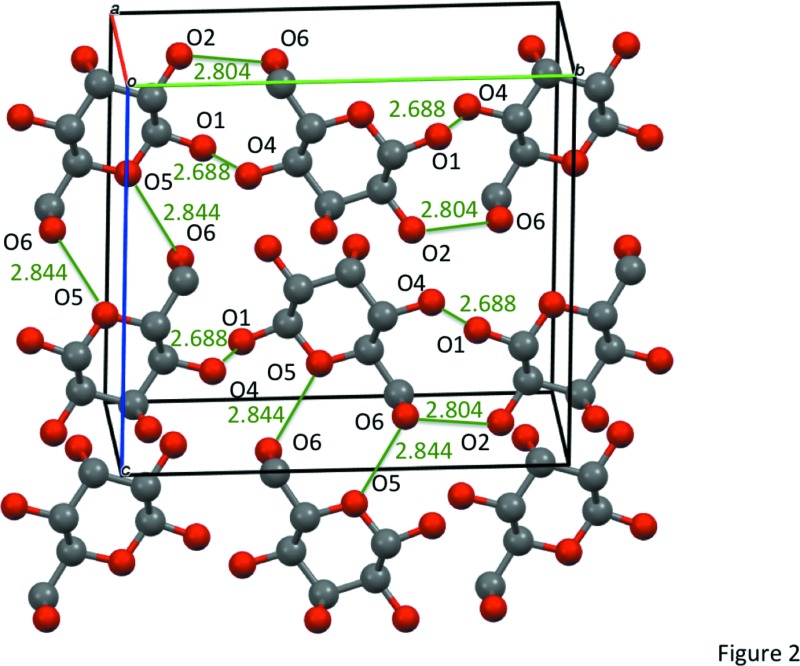
Part of the crystal structure of the title compound with hydrogen-bonding network represented as light green dashed lines, viewed down the tilted a axis. The hydrogen atoms are omitted for clarity.

**Fig. 3.**
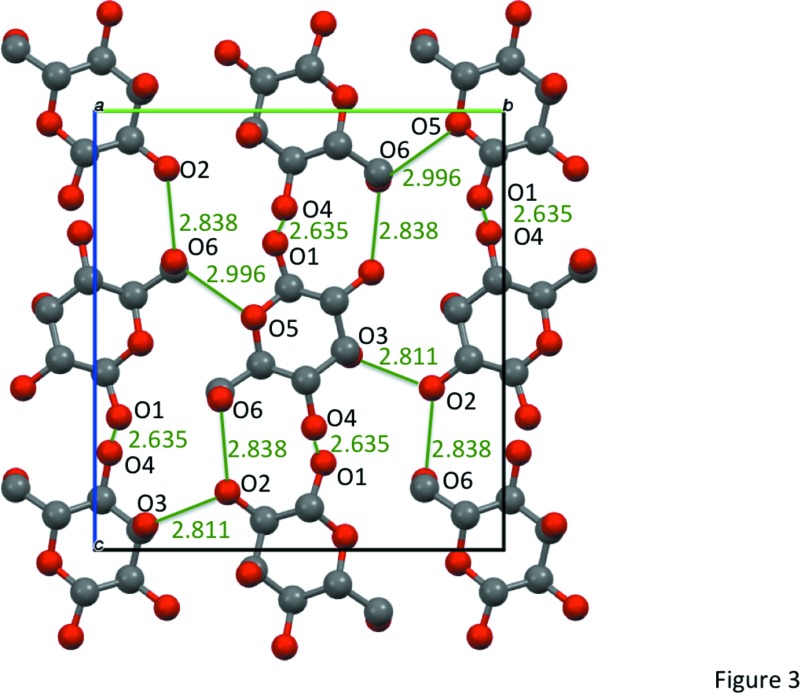
Part of the crystal structure of the chiral β-D-allose (Kroon-Batenburg et al., 1984) with hydrogen-bonding network represented as light blue dashed lines, viewed down the a axis. The hydrogen atoms are omitted for clarity.

ORTEP . DOI: 10.1107/S2056989015000353/is5386fig1.tif
*ORTEP* view of the title compound with the atom-labeling scheme. The thermal ellipsoids of all non-hydrogen atoms are drawn at the 50% probability level. H atoms are shown as small spheres of arbitrary radius.

a . DOI: 10.1107/S2056989015000353/is5386fig2.tif
Part of the crystal structure of the title compound with hydrogen-bonding network represented as light green dashed lines, viewed down the tilted *a* axis. The hydrogen atoms are omitted for clarity.

d et al. a . DOI: 10.1107/S2056989015000353/is5386fig3.tif
Part of the crystal structure of the chiral β-d-allose (Kroon-Batenburg *et al.*, 1984) with hydrogen-bonding network represented as light blue dashed lines, viewed down the *a* axis. The hydrogen atoms are omitted for clarity.

CCDC reference: 1037204

Additional supporting information:  crystallographic information; 3D view; checkCIF report

## Figures and Tables

**Table 1:** Hydrogen-bond geometry (, )

*D*H*A*	*D*H	H*A*	*D* *A*	*D*H*A*
O1H1*A*O4^i^	0.82	1.88	2.6884(16)	171
O2H2*A*O6^i^	0.82	1.99	2.8044(16)	172
O3H3*A*O2^ii^	0.82	1.94	2.7494(16)	169
O4H4*A*O1^iii^	0.82	1.94	2.7384(16)	163
O6H6*A*O5^iv^	0.82	2.03	2.8439(15)	171

Symmetry codes: (i) 
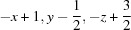
; (ii) 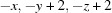; (iii) 
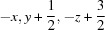
; (iv) 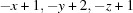.

## supplementary crystallographic information

### S1. Comment

Orientations of three hydrogen atoms (H1A, H2A, H4A) located on three equatorial OH groups at C-1, C-2 and C-4 positions are perpendicular to the pyranose ring. A hydrogen atom (H3A) on the axial OH group at C-3 position is located along to the radial direction. Therefore, it is inconvenient to obtain the intramolecular hydrogen bonding. Only a weak intramolecular hydrogen bonding (O4—H4A···O3) is found in the racemic β-*D,L*-allose, with long interatomic distance (H4A···O3; 2.49 Å) and the small bond angle (O4—H4A···O3; 106°). In the case of the chiral β-*D*-allose, there are five hydrogen bonding (O1—H1A···O4, O2—H2A···O6, O3—H3A···O2, O4—H4A···O1, O6—H6A···O5) observed between two adjacent *D*-allose molecules (Kroon-Batenburg *et al.*, 1984). These five hydrogen bondings are also observed in the racemic β-*D,L*-allose with a same corresponding sequential number. Three of them (O1—H1A···O4^i^, O2—H2A···O6^i^ and O4—H4A···O1^iii^; Table 1) are used for creating the hydrogen bonding network between two adjacent *D*-molecules or *L*-molecules, forming a homochiral layer parallel to the *ab*-plane. The remaining hydrogen bonds (O3—H3A···O2^ii^ and O6—H6A···O5^iv^; Table 1) are used for connecting between the *D*- and *L*-allose molecules. An example of the unit cell volume of racemic compound less than that of chiral one was also found in the case of racemic *D,L*-arabinose (*V* = 596.516 Å^3^ at 175 K; Longchambon *et al.*, 1985) and chiral *L*-arabinose (*V* = 598.661 Å^3^ at 123 K; Takagi *et al.*, 1977).

### S2. Experimental

*D*-Allose and *L*-allose were biosynthesized from *D*-psicose and *L*-psicose using *L*-rhammose isomerase (Menavuvu *et al.*, 2006; Morimoto *et al.*, 2006) and *L*-ribose isomerase (Shimonishi *et al.*, 1996; Morimoto *et al.*, 2013), respectively. Equimolar mixture of *D*-allose and *L*-allose was dissolved in water to give 15 wt% solution, and then it was kept at 30 °C. After two days, small crystals appeared and they were grown at 25 °C for two weeks yielded prism-shaped crystals of sufficient size. Melting point of the obtained crystals was confirmed to be 181 °C, which was 30–35 °C higher than the melting point of β-*D*-allose.

### S3. Refinement

H atoms bounded to methine-type C (H1B, H2B, H3B, H4B, H5A) were positioned geometrically and refined using a riding model with C—H = 0.98 Å and *U*_iso_(H) = 1.2*U*_eq_(C). H atoms bounded to methylene-type C (H6B, H6C) were positioned geometrically and refined using a riding model with C—H = 0.97 Å and *U*_iso_(H) = 1.2*U*_eq_(C). H atoms bounded to O (H1A, H2A, H3A, H4A, H6A) were positioned geometrically and refined using a riding model with O—H = 0.82 Å and *U*_iso_(H) = 1.2*U*_eq_(O), allowing for free rotation of the OH groups.

### Crystal data

**Table:**

C_6_H_12_O_6_	*F*(000) = 384.00
*M**_r_* = 180.16	*D*_x_ = 1.618 Mg m^−^^3^
Monoclinic, *P*2_1_/*c*	Cu *K*α radiation, λ = 1.54187 Å
Hall symbol: -P 2ybc	Cell parameters from 11709 reflections
*a* = 4.98211 (10) Å	θ = 3.5–68.2°
*b* = 12.5624 (3) Å	µ = 1.29 mm^−^^1^
*c* = 11.8156 (3) Å	*T* = 295 K
β = 91.1262 (14)°	Block, colorless
*V* = 739.36 (3) Å^3^	0.10 × 0.10 × 0.10 mm
*Z* = 4	

### Data collection

**Table:**

Rigaku R-AXIS RAPID diffractometer	1232 reflections with *F*^2^ > 2σ(*F*^2^)
Detector resolution: 10.000 pixels mm^-1^	*R*_int_ = 0.075
ω scans	θ_max_ = 68.2°
Absorption correction: multi-scan (*ABSCOR*; Higashi, 1995)	*h* = −6→6
*T*_min_ = 0.687, *T*_max_ = 0.879	*k* = −15→15
12963 measured reflections	*l* = −14→14
1350 independent reflections	

### Refinement

**Table:**

Refinement on *F*^2^	Hydrogen site location: inferred from neighbouring sites
*R*[*F*^2^ > 2σ(*F*^2^)] = 0.037	H-atom parameters constrained
*wR*(*F*^2^) = 0.102	*w* = 1/[σ^2^(*F*_o_^2^) + (0.0463*P*)^2^ + 0.3535*P*] where *P* = (*F*_o_^2^ + 2*F*_c_^2^)/3
*S* = 1.07	(Δ/σ)_max_ = 0.001
1350 reflections	Δρ_max_ = 0.37 e Å^−^^3^
115 parameters	Δρ_min_ = −0.22 e Å^−^^3^
0 restraints	Extinction correction: *SHELXL2013* (Sheldrick, 2015)
Primary atom site location: structure-invariant direct methods	Extinction coefficient: 0.0159 (15)
Secondary atom site location: difference Fourier map	

### Special details

**Table:**

Refinement. Refinement was performed using all reflections. The weighted *R*-factor (*wR*) and goodness of fit (*S*) are based on *F*^2^. *R*-factor (gt) are based on *F*. The threshold expression of *F*^2^ > 2.0 σ(*F*^2^) is used only for calculating *R*-factor (gt).

### Fractional atomic coordinates and isotropic or equivalent isotropic displacement parameters (Å^2^)

**Table:**

	*x*	*y*	*z*	*U*_iso_*/*U*_eq_	
O1	0.1621 (3)	0.79703 (8)	0.67894 (10)	0.0283 (3)	
O2	0.1676 (3)	0.85269 (9)	0.91826 (9)	0.0311 (4)	
O3	−0.0213 (2)	1.05723 (8)	0.88005 (9)	0.0244 (3)	
O4	0.3426 (3)	1.21319 (8)	0.80258 (10)	0.0270 (3)	
O5	0.2673 (2)	0.96785 (8)	0.63757 (8)	0.0219 (3)	
O6	0.6115 (3)	1.14807 (9)	0.56175 (9)	0.0282 (3)	
C1	0.1454 (3)	0.90015 (11)	0.71937 (12)	0.0211 (4)	
C2	0.2848 (3)	0.91736 (11)	0.83377 (12)	0.0207 (4)	
C3	0.2552 (3)	1.03366 (11)	0.86734 (12)	0.0195 (4)	
C4	0.3680 (3)	1.10357 (11)	0.77418 (12)	0.0189 (4)	
C5	0.2263 (3)	1.07910 (11)	0.66196 (12)	0.0197 (4)	
C6	0.3259 (4)	1.14427 (12)	0.56431 (13)	0.0259 (4)	
H1A	0.3191	0.7774	0.6812	0.0340*	
H1B	−0.0442	0.9196	0.7254	0.0254*	
H2A	0.2377	0.7936	0.9180	0.0373*	
H2B	0.4758	0.8998	0.8280	0.0248*	
H3A	−0.0440	1.0847	0.9421	0.0293*	
H3B	0.3529	1.0470	0.9387	0.0234*	
H4B	0.5592	1.0872	0.7668	0.0226*	
H4A	0.1849	1.2268	0.8151	0.0324*	
H5A	0.0336	1.0920	0.6699	0.0237*	
H6A	0.6640	1.1146	0.5069	0.0339*	
H6C	0.2577	1.1140	0.4939	0.0311*	
H6B	0.2565	1.2162	0.5702	0.0311*	

### Atomic displacement parameters (Å^2^)

**Table:**

	*U*^11^	*U*^22^	*U*^33^	*U*^12^	*U*^13^	*U*^23^
O1	0.0289 (7)	0.0177 (6)	0.0386 (7)	−0.0020 (5)	0.0058 (5)	−0.0067 (5)
O2	0.0465 (8)	0.0190 (6)	0.0283 (6)	0.0071 (5)	0.0162 (5)	0.0067 (5)
O3	0.0230 (6)	0.0284 (6)	0.0222 (6)	0.0063 (5)	0.0066 (4)	−0.0025 (5)
O4	0.0271 (6)	0.0147 (6)	0.0393 (7)	−0.0005 (4)	0.0044 (5)	−0.0042 (5)
O5	0.0292 (7)	0.0174 (6)	0.0193 (6)	−0.0019 (4)	0.0049 (5)	−0.0011 (4)
O6	0.0303 (7)	0.0300 (7)	0.0247 (6)	−0.0063 (5)	0.0089 (5)	−0.0044 (5)
C1	0.0232 (8)	0.0154 (8)	0.0250 (8)	−0.0019 (6)	0.0047 (6)	−0.0016 (6)
C2	0.0233 (8)	0.0182 (8)	0.0207 (8)	0.0035 (6)	0.0054 (6)	0.0030 (6)
C3	0.0202 (8)	0.0195 (8)	0.0187 (7)	0.0025 (6)	−0.0005 (6)	−0.0010 (6)
C4	0.0187 (8)	0.0139 (7)	0.0240 (8)	0.0013 (6)	0.0013 (6)	−0.0023 (6)
C5	0.0199 (8)	0.0165 (8)	0.0229 (8)	0.0017 (6)	0.0022 (6)	0.0002 (6)
C6	0.0282 (9)	0.0251 (8)	0.0245 (8)	0.0008 (6)	0.0025 (6)	0.0052 (7)

### Geometric parameters (Å, º)

**Table:**

O1—C1	1.3837 (18)	O1—H1A	0.820
O2—C2	1.4216 (19)	O2—H2A	0.820
O3—C3	1.4199 (18)	O3—H3A	0.820
O4—C4	1.4236 (18)	O4—H4A	0.820
O5—C1	1.4314 (18)	O6—H6A	0.820
O5—C5	1.4423 (18)	C1—H1B	0.980
O6—C6	1.425 (2)	C2—H2B	0.980
C1—C2	1.523 (2)	C3—H3B	0.980
C2—C3	1.522 (2)	C4—H4B	0.980
C3—C4	1.524 (2)	C5—H5A	0.980
C4—C5	1.521 (2)	C6—H6C	0.970
C5—C6	1.507 (2)	C6—H6B	0.970
			
C1—O5—C5	112.16 (11)	C6—O6—H6A	109.474
O1—C1—O5	107.10 (12)	O1—C1—H1B	108.857
O1—C1—C2	114.20 (12)	O5—C1—H1B	108.860
O5—C1—C2	108.84 (12)	C2—C1—H1B	108.859
O2—C2—C1	110.83 (12)	O2—C2—H2B	109.452
O2—C2—C3	108.80 (12)	C1—C2—H2B	109.449
C1—C2—C3	108.83 (12)	C3—C2—H2B	109.458
O3—C3—C2	109.09 (12)	O3—C3—H3B	109.847
O3—C3—C4	109.17 (12)	C2—C3—H3B	109.848
C2—C3—C4	109.02 (12)	C4—C3—H3B	109.842
O4—C4—C3	110.57 (12)	O4—C4—H4B	108.392
O4—C4—C5	111.04 (12)	C3—C4—H4B	108.388
C3—C4—C5	109.98 (12)	C5—C4—H4B	108.388
O5—C5—C4	107.75 (11)	O5—C5—H5A	108.775
O5—C5—C6	108.87 (12)	C4—C5—H5A	108.778
C4—C5—C6	113.79 (12)	C6—C5—H5A	108.780
O6—C6—C5	112.25 (13)	O6—C6—H6C	109.153
C1—O1—H1A	109.467	O6—C6—H6B	109.157
C2—O2—H2A	109.472	C5—C6—H6C	109.152
C3—O3—H3A	109.470	C5—C6—H6B	109.146
C4—O4—H4A	109.476	H6C—C6—H6B	107.880
			
C1—O5—C5—C4	63.92 (13)	C1—C2—C3—C4	−56.31 (14)
C1—O5—C5—C6	−172.24 (10)	O3—C3—C4—O4	60.68 (14)
C5—O5—C1—O1	171.24 (10)	O3—C3—C4—C5	−62.31 (13)
C5—O5—C1—C2	−64.83 (13)	C2—C3—C4—O4	179.76 (11)
O1—C1—C2—O2	−61.24 (16)	C2—C3—C4—C5	56.77 (14)
O1—C1—C2—C3	179.15 (11)	O4—C4—C5—O5	178.40 (10)
O5—C1—C2—O2	179.15 (10)	O4—C4—C5—C6	57.59 (15)
O5—C1—C2—C3	59.54 (14)	C3—C4—C5—O5	−58.88 (14)
O2—C2—C3—O3	−58.04 (14)	C3—C4—C5—C6	−179.69 (10)
O2—C2—C3—C4	−177.17 (10)	O5—C5—C6—O6	−74.12 (14)
C1—C2—C3—O3	62.82 (14)	C4—C5—C6—O6	46.06 (16)

### Hydrogen-bond geometry (Å, º)

**Table:**

*D*—H···*A*	*D*—H	H···*A*	*D*···*A*	*D*—H···*A*
O1—H1*A*···O4^i^	0.82	1.88	2.6884 (16)	171
O2—H2*A*···O6^i^	0.82	1.99	2.8044 (16)	172
O3—H3*A*···O2^ii^	0.82	1.94	2.7494 (16)	169
O4—H4*A*···O1^iii^	0.82	1.94	2.7384 (16)	163
O4—H4*A*···O3	0.82	2.49	2.8333 (15)	106
O6—H6*A*···O5^iv^	0.82	2.03	2.8439 (15)	171

Symmetry codes: (i) −*x*+1, *y*−1/2, −*z*+3/2; (ii) −*x*, −*y*+2, −*z*+2; (iii) −*x*, *y*+1/2, −*z*+3/2; (iv) −*x*+1, −*y*+2, −*z*+1.
