# Supplementary figures and images for: Crystal structure of β-d,l-allose
Source: Acta Crystallogr E Crystallogr Commun. 2015 Jan 31;71(Pt 2):o139. doi: 10.1107/S2056989015000353 (PMC4384602; doi:10.1107/S2056989015000353)

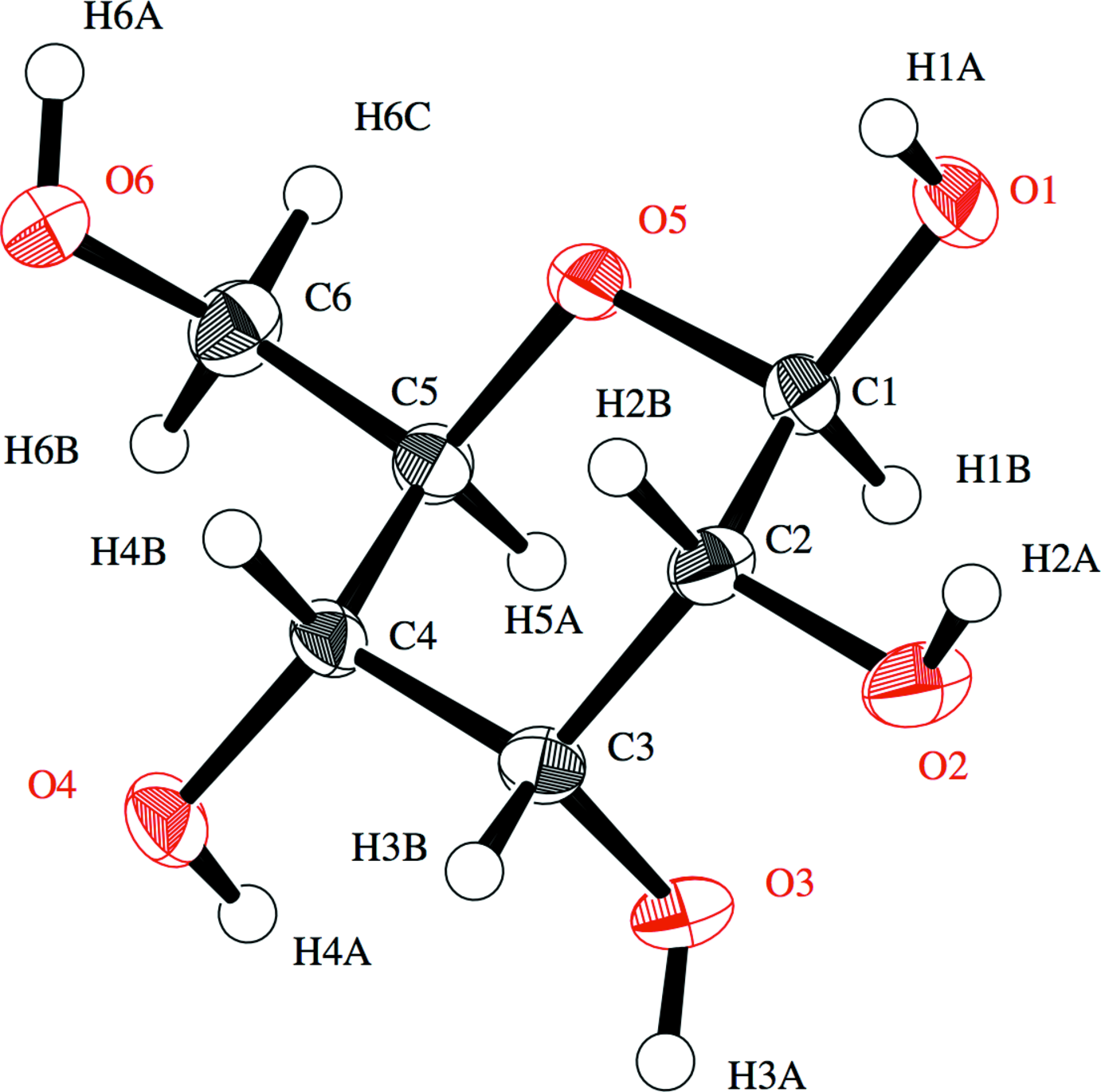

Supplement: Supplementary file 3 [file e-71-0o139-fig1.tif]

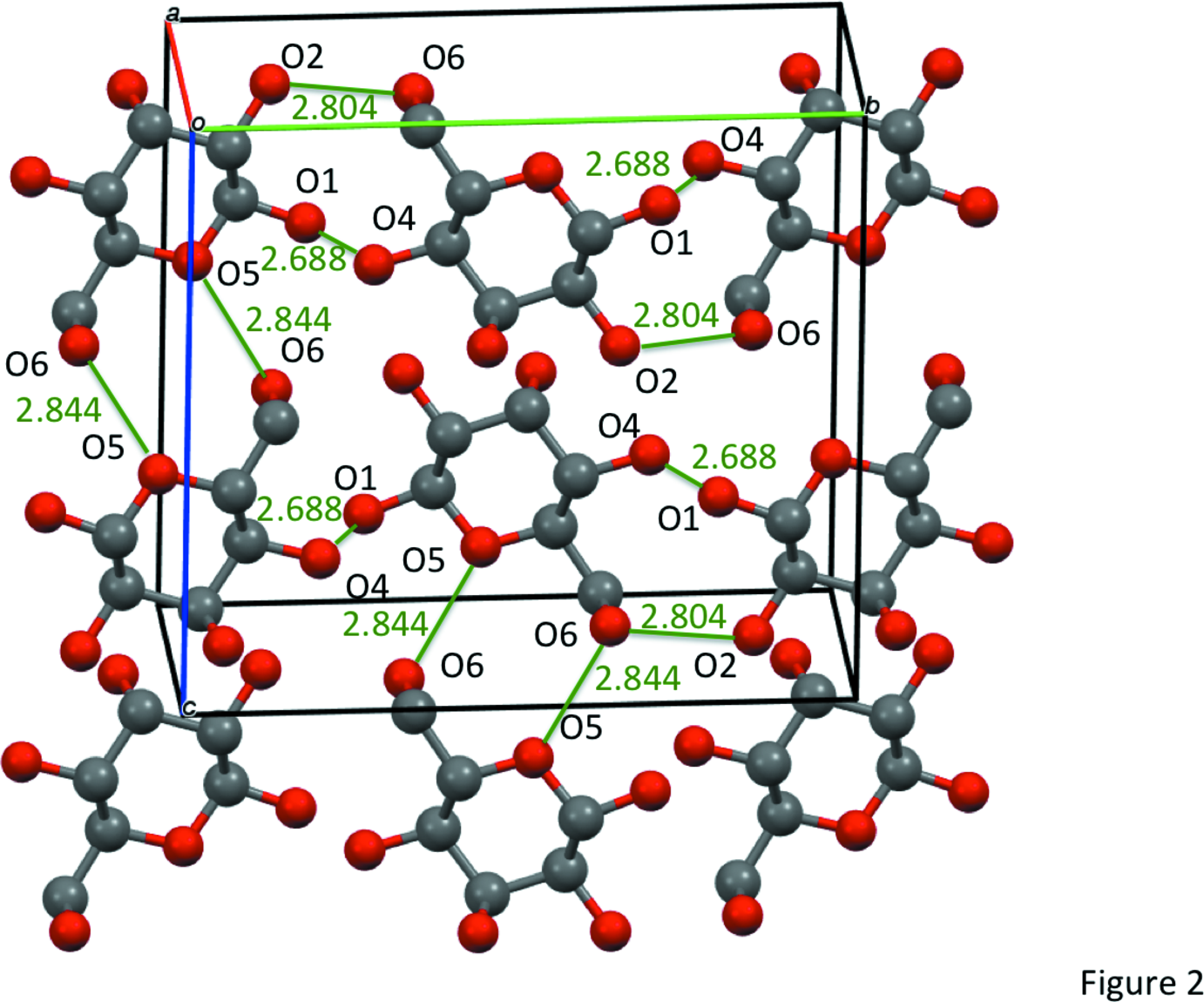

Supplement: Supplementary file 4 [file e-71-0o139-fig2.tif]

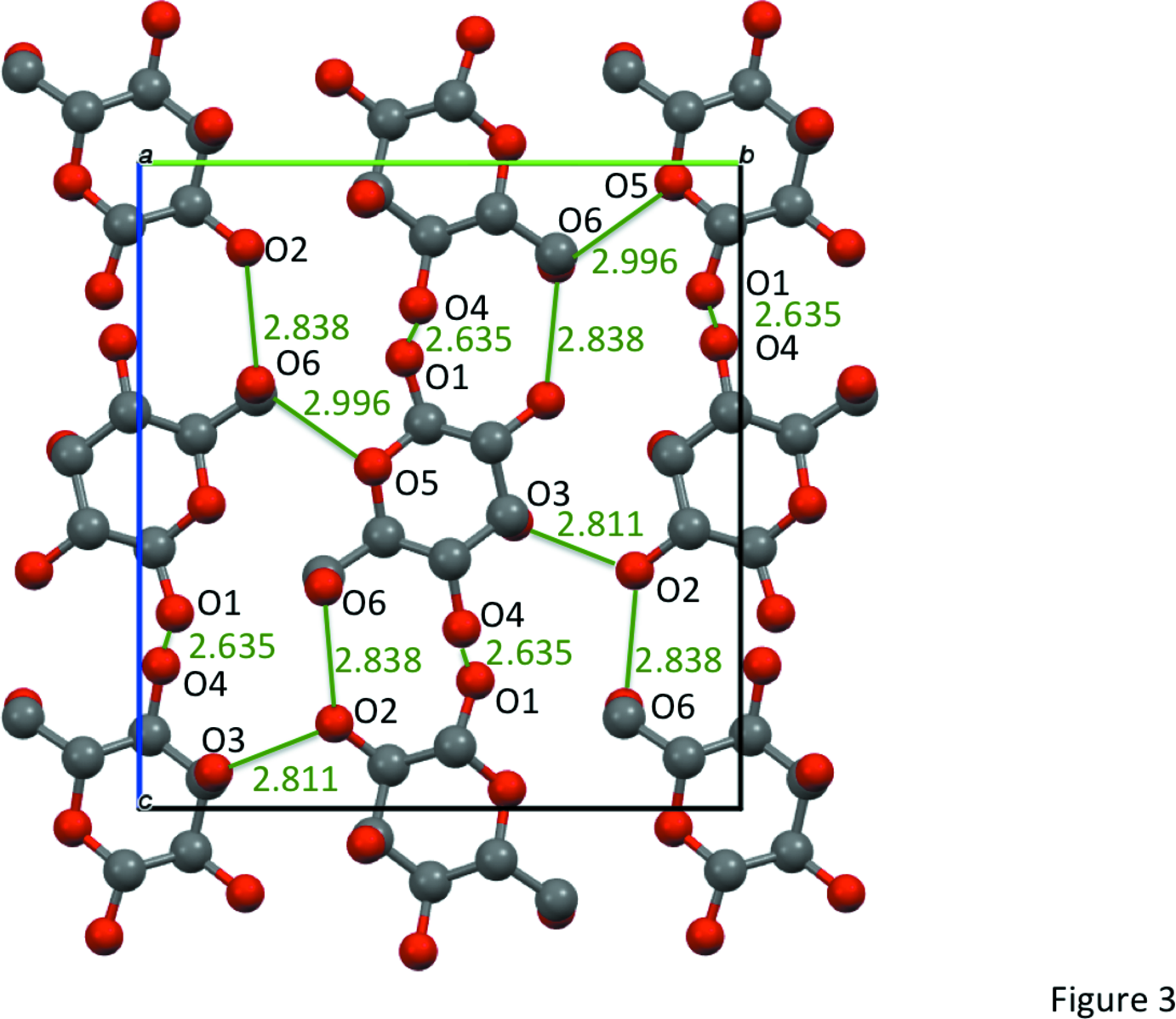

Supplement: Supplementary file 5 [file e-71-0o139-fig3.tif]
